# Are Healthcare Resource Utilization Patterns for Pain Management Specific to Post-Acute COVID-19 Syndrome? A Study of Survivors from the First French Pandemic Wave

**DOI:** 10.3390/jcm13247680

**Published:** 2024-12-17

**Authors:** Mikhail Dziadzko, Manon Belhassen, Eric Van Ganse, Claire Marant-Micallef, Valeria Martinez, Frederic Aubrun

**Affiliations:** 1 Département d’Anesthésie-Réanimation, Douleur, Hôpital de la Croix Rousse, Hospices Civils de Lyon, 69004 Lyon, France; 2 PELyon, 69007 Lyon, France; 3 Service d’Anesthésie Réanimation Chirurgicale, Hôpital Raymond Poincaré, Assistance Publique Hôpitaux de Paris, 92380 Garches, France

**Keywords:** COVID-19, epidemiology, healthcare resource utilization, pain management, post-acute COVID-19 syndrome

## Abstract

**Objectives:** Chronic pain is a common symptom in Post-Acute COVID-19 Syndrome (PACS), affecting 11–60% of patients, but the link between COVID-19 and chronic pain remains unclear. This study assesses healthcare resource utilization (HRU) for pain management among French COVID-19 survivors, using the National French Claims Database (SNDS). We analyzed medical consultations, rehabilitation services, diagnostic procedures, and medication dispensing to identify PACS-related pain patterns and their impact on the healthcare system. **Methods:** The cohort included 68,822 patients hospitalized during the first COVID-19 wave (March–June 2020), with 13,939 ICU survivors. HRU was assessed for six months pre- and post-hospitalization in four areas: (1) medical consultations and rehabilitation; (2) pain-related medication dispensing; (3) neuropathic diagnostic procedures; (4) hospital admissions for chronic pain. A post–pre ratio (PP-Ratio) compared post-COVID to pre-COVID HRU. **Results:** Significant changes in HRU were observed, particularly for ICU survivors. Neurology consultations (PP-Ratio 1.41) and outpatient physical therapy (PP-Ratio 1.69) increased. Dispensing of strong opioids, antiepileptics, anxiolytics, and hypnotics rose, while NSAID use decreased. Hospitalizations for chronic pain also increased (PP-Ratio 1.52). Similar trends were seen among ICU survivors, with notable increases in opioid and antiepileptic use. No distinct PACS-related pain patterns emerged. **Conclusions:** Non-specific increases in HRU for pain management were found following COVID-19 hospitalization, likely due to disease severity and ICU care rather than PACS-related chronic pain. Further research is needed to explore long-term pain outcomes in this population.

## 1. Introduction

Post-hospital healthcare resource use (HRU) among patients admitted for acute COVID-19 infection is frequently associated with the initiation of new care needs following discharge [1]. Multiple studies and reports indicate an elevated burden of various pain-related symptoms following acute Coronavirus-19 (COVID-19) infection, recognized as Post-Acute COVID-19 Syndrome (PACS) [2,3]. Chronic pain is a common feature of PACS, with prevalence estimates ranging from 11% to 60% [4]. Persistent symptoms, including headaches, musculoskeletal pain, and abdominal pain, have been reported after SARS-CoV-2 infection, notably following discharge from hospitals and intensive care units [5,6,7].

The initial wave of the French pandemic was characterized by a high rate of hospitalizations, ICU admissions, and aggressive therapy [8]. Simultaneously, the reorganization and reallocation of medical resources have altered the post-discharge follow-up of such patients and the functioning of specialized structures, such as French Chronic Pain Structures [9]. These structures involve various medical specialties, such as neurology, rheumatology, psychiatry, and physical medicine and rehabilitation. We hypothesize that analyzing changes in the rates of medical consultations, rehabilitation services, diagnostic procedures, medication prescriptions, and hospitalizations may help identify new patterns of chronic pain related to PACS.

While observational studies indicate a significant increase in chronic pain conditions among COVID-19 convalescents, there is limited knowledge regarding the impact and potential pain-related burden in this population. It is plausible that there will be growth in the consumption of pain medications and an increased demand for the services of pain specialists. Leveraging the National French Claims Database (SNDS), we aimed to describe and analyze the healthcare resource utilization (HRU) associated with pain management in patients discharged after COVID-19. Additionally, we compare the HRU post-COVID-19 discharge to that of the same patients before the SARS-CoV-2 pandemic.

## 2. Materials and Methods

### 2.1. Study Design and Population

We performed a secondary analysis on the cohort of 68,822 patients hospitalized for COVID-19 during the first pandemic wave (1 March to 30 June 2020), with a subgroup of 13,939 ICU survivors, identified from our previously reported study [1].

**Figure 1:**
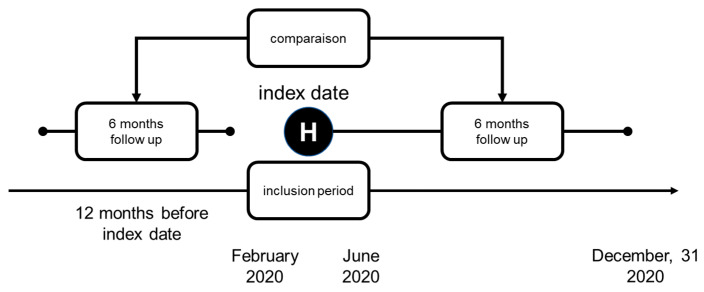
Study design. H: date of discharge after hospitalization for COVID-19 infection = index date.

Briefly, this is a retrospective population-based cohort study, conducted through the Système National des Données de Santé (SNDS), the French National Health System claims. Access to the SNDS was granted through the French Health Data Hub, which also serves as the regulatory body overseeing the use of health data for research purposes (https://health-data-hub.fr, accessed on 8 July 2021). All adult patients hospitalized for COVID-19 between the first French pandemic wave between 1 February and 30 June 2020 and alive at their discharge date (index date) were identified, with follow-up 6 months after discharge. The discharge diagnosis codes of COVID-19 (U07.10, U07.11, U07.14, and U07.15) were used for identification. Patients who expired during hospitalization and within 30 days following hospital discharge and those who were pregnant, as identified between 1 February 2019 and 31 December 2020, were excluded. To describe changes in HRU, the identified patients were followed up during a 6-month period, within one year anteriorly to the index date (Figure 1). Patients with no HRU data for 12 months preceding the index date were excluded.

### 2.2. Endpoints

Following the Delphi process, experts in pain management (VM, FA, MD) defined four categories of HRU likely to be impacted by either new onset pain or exacerbation of pre-existing chronic pain before pandemics: (1) physician consultations (general practitioners and physicians traditionally involved in chronic pain management in France, such as neurologists, rheumatologists, physical medicine specialists, psychiatrists, and pain specialists) and rehabilitation; (2) dispensed medications related to pain management; (3) instrumental investigations potentially related to nerve injuries; and (4) hospital admissions for chronic pain, as detailed in the Table 1. The selection of these categories and endpoints was achieved through a single round of expert discussion and consensus, leading to agreement on the final list of endpoints. The rationale and selection process were guided by the evidence available at the time of study conception [10,11], expert clinical judgment regarding pain management practices in France, and the feasibility of extracting relevant HRU items from the SNDS. For every item in each category, the SNDS database contains all nonhospital reimbursed healthcare expenditures along with timestamps. The SNDS does not contain information on behavioral and clinical characteristics or laboratory results.

### 2.3. Statistical Analysis

For each item, we calculated the proportion of patients having at least one HRU during the follow-up period and the rate of HRU (number of HRU per month during the follow-up period). Next, a post–pre- ratio (PP Ratio) was defined for each domain and for all items as the ratio of the post-COVID percentage of users or HRU rate divided by the pre-COVID percentage of the user for the HRU rate.

Arbitrarily, the major changes in HRU occurred if 2 conditions were simultaneously met: the post-COVID percentage of patients using the domain or item was ≥1% of the study population AND the post/pre ratio (PP Ratio) of the percentages of the domain or item was ≥1.2 or ≤0.8.

The monthly rate of HRU (number of uses per month) was calculated with its PP Ratio with a 95% confidence interval. A PP Ratio indicates the increase (PP Ratio > 1) or decrease (<1) in the number of patients with HRU or the frequency of HRU use.

The concept of the pre–post-ratio enables the detection of variations in HRU in two ways: by observing increases (new consummation) or decreases in the proportion of patients incurring resource use changes, and by noting changes in the frequency of use for each studied item. For example, we may expect exacerbation of chronic conditions after COVID-19 exposure if the PP Ratio remains unchanged but the RR ratio increases (indicating more frequent use of the same resources by the same patients).

Descriptive statistics were used. Data are expressed as number and frequencies, means, and standard deviation or as median [interquartile range], means, and 95% confidence interval for PP-Ratio Rates. All statistical analyses were performed using SAS (SAS Institute, Cary, NC, USA), version 9.4.

## 3. Results

Between 1 February 2020 and 30 June 2020, a total of 90,025 patients were hospitalized with a primary diagnostic code of COVID-19. Following the application of successive exclusion criteria, the final study population comprised 68,822 patients [1]. Among those, 13,939 were hospitalized in the Intensive Care Unit (ICU). The median age was 66 [52 to 80] years, and there were 32,372 (47%) women. The median follow-up duration was 183 days [31 to 184].

Within the studied population of 68,822 patients, major HRU changes occurred in all domains (Table 2). 

### 3.1. Medical Consultations and Rehabilitation After Discharge

In this domain, major HRU changes were found for non-coordinated visits (specialized consultations without referrals), with the increasing number and frequency of HRU. In the sub-population experiencing ICU admission, major HRU changes were noted for non-coordinated consultations (increase) and rheumatology specialists (decrease).

A marked rise was observed in outpatient rehabilitation care and occupational therapy, with a consistent increase in the number of patients, without major changes in the rate of visits.

### 3.2. Instrumental Diagnostics

There was a substantial increase in HRU related to instrumental diagnostics limited to peripheral nerve sonography and electroneuromyography, particularly evident among ICU survivors.

### 3.3. HRU and Medication Dispensing

In the overall population, a major increase in HRU occurred for strong opioids, antiepileptics with chronic pain modulation activity, anxiolytics, hypnotics, and vitamin D, accompanied by a decrease in non-steroidal anti-inflammatory drugs (NSAIDs). In the post-ICU patient population, similar HRU changes were noted for antiepileptics and antidepressants with chronic pain modulation activity, strong opioids, other analgesics, psychoactive drugs (antidepressants, anxiolytics, hypnotics), and vitamin D.

### 3.4. Hospitalizations

Major HRU changes in all-cause hospitalizations and particularly for hospitalization for chronic pain were observed, both in the overall and ICU survivor populations.

**Figure 2:**
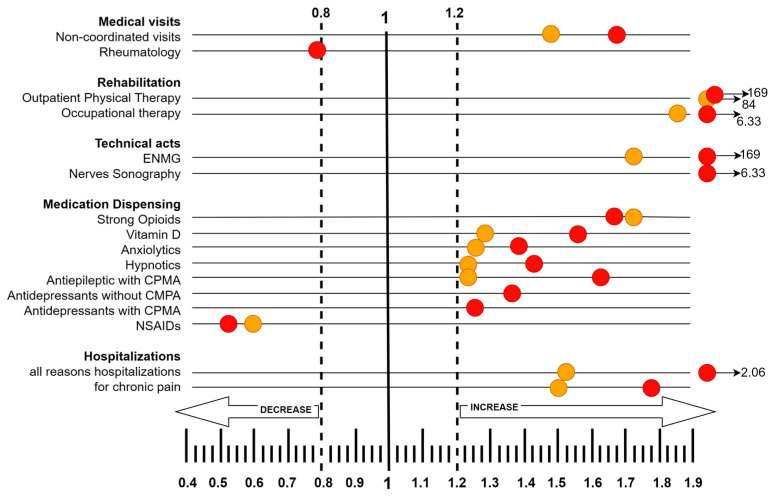
Summary of Major Changes in Healthcare Resource Utilization Related to Pain Management Post-COVID-19 Discharge). Footnote: ENMG—Electroneuromyogram; CPMA—chronic pain modulation activity; NSAIDs—non-steroidal anti-inflammatory drugs. Orange dots—whole population; Red dots—patients with ICU stay; numbers after arrows indicate outlier ratios. Scale is a post–pre ratio for Healthcare Resource Use.

No individual item from any domain displayed major changes in the rate of HRU use (95% confidence interval range includes a negative value). Summary data for major changes (regarding ≥1% of population post discharge and having ≥20% HRU changes) are presented in Figure 2.

## 4. Discussion

We found heterogeneous and nonspecifically increased HRU following the first French pandemic wave, including increased medical consultations, particularly in neurology for ICU survivors, a significant rise in outpatient rehabilitation, and an important escalation in instrumental diagnostics, particularly in the intensive care sub-cohort. Medication dispensing patterns showed marked changes, with increased utilization of several pain-related medications and unexpectedly small changes in the prescription of medications targeting chronic pain modulation [12]. Increased hospitalizations for chronic pain were found. No specific post-COVID-19 pain-related patterns were identified.

The reported prevalence of chronic pain in the French population is about 30% (approximately 22 million people), and 5% of the adult population presents moderate to severe chronic pain with neuropathic characteristics [13,14]. As such, one might expect considerable relapse, exacerbation, or the occurrence of new-onset neuropathic and/or oncoplastic pain in the studied population [12]. Although the pooled data derived from SNDS do not allow for analyses of clinical diagnoses, particular patterns of chronic pain-associated HRU, especially changes in the rate of HRU, would be expected. Recent evidence on the persistent pain syndrome in COVID-19 survivors may definitely increase the disease burden in the population [3,6,15].

We focused on the HRU commonly involved in the French model of chronic pain management. Patients are typically oriented by general practitioners toward pain specialists (algologists) or physicians with advanced training in pain management (mainly, but not exclusively, neurologists, rheumatologists, physical medicine specialists, psychiatrists, and rehabilitation medicine specialists), with associated investigations and medication prescriptions [16,17]. In addition, we looked for several specific items, such as electromyography, peripheral nerve sonography, antiseizure and antidepressant medication with chronic pain modulation activity, and midodrine prescriptions, following reports on the potential role of the SARS-CoV-2 pathogen in peripheral and central neuropathy and dysautonomia disorders [18,19,20,21]. We considered both the new consumers of HRU (increase in percentage and PP Ratio > 1) and augmentation of the frequency of HRU (monthly rate, PP rate ratio > 1).

We did not observe an increase in pain specialist consultations. Such specialists usually practice in chronic pain structures (CPSs), offering multidisciplinary pain care. Before the COVID-19 outbreak, there were over 240 French CPSs in operation, serving an estimated annual volume of 400,000 patients. However, the first COVID-19 wave necessitated the reallocation of over 40% of pain specialists to other specialties. Additionally, more than 70% of in-person appointments for chronic pain patients were cancelled [9]. Despite the impact of the pandemic on the functioning of CPSs, we observed a 1% increase in the proportion of patients with at least one hospitalization for chronic pain; however, this represents 717 out of 68,822 (1.04%) in the overall population. Marked changes in physical and occupational therapy new consultations without increased frequency may strongly suggest a pandemic effect rather than a SARS-CoV-2 specific effect.

We did observe an increase in the new dispensation of opioids and psychotropic medications, more importantly in the ICU sub-population; however, no increase in the frequency of dispensation of opioids, psychoactive medications, or antiseizure/antidepressants used for chronic pain was noted. During the pandemic period, a derogatory authorization was granted to French pharmacists, allowing for opiates and psychotropic medications for patients with an expired prescription to maintain therapeutic equilibrium for pain and psychological conditions during the pandemic [22]. Again, we explain our findings by the pandemic effect and also by the effect of exposure to aggressive care in the ICU or in ICU-like units, created during overflowing admissions of the critically ill [8].

A reduction in the consumption of NSAIDs (up to −75%) was probably linked to the warning on the use of anti-inflammatory drugs issued early by the French health authorities for a potential risk of aggravating COVID-19. The dispensing of ibuprofen was virtually halted following messages from health authorities, while prescriptions for paracetamol reached up to 1 million patients per day [23,24,25]. There was a discrepancy between the precautionary principle regarding the unsafe use of NSAIDs in cases of infection and the reality that chronic treatment with NSAIDs for inflammatory conditions should be continued [26,27,28,29,30,31].

All drugs reducing anxiety and mood disorders saw their consumption increase over the period studied, both in terms of new patients and the frequency of dispensation. The most prevalent reported psychiatric symptoms after COVID-19 infection, from the most to the least reported, were anxiety, depression, post-traumatic stress disorder (PTSD), poor sleep quality, somatic symptoms, and cognitive deficits [32]. These results are in line with published observations on the use of prescription drugs in France in 2020 and 2021 during the COVID-19 epidemic [33]. The increase in drug consumption reflects the visible part of the major psychological impact of the epidemic on the French population and its social, professional, and economic consequences [34].

A significant increase in vitamin D consumption certainly corresponds to past publications or recommendations published during the first wave about the risk of vitamin D deficiency. Liu et al. performed a systematic review between 2018 and 2021 about medications that may alter the risk and severity of COVID-19 infections [35] and noted that deficiency in vitamin D has been linked to high rates of COVID-19 infections [36].

The study presents several limitations. We focused exclusively on patients hospitalized for COVID-19, omitting those with milder forms, and no data on SARS-CoV-2 rapid tests was available, limiting data collection only to ICD codes associated with COVID. The management of severe cases during the first wave may contribute to a heightened prevalence of post-intensive care syndrome, challenging its distinction from potential PACS.

We employed arbitrarily defined thresholds (1% for population changes and 20% for ratio changes). These thresholds were applied in our exhaustive population-based study. Consequently, even a small percentage change may hold significance, particularly if it pertains to a critical aspect of public health, such as pain management.

A negative result was reported. During the initial wave of the pandemic, when specific information on severe COVID-19 or post-COVID syndrome was scarce, no positive control group of drugs or other healthcare resource utilization (HRU) likely affected by COVID-19 was chosen for comparison alongside pain medications, which were the primary focus of the analysis.

Utilizing the French National Health System claims database hinders the identification of comprehensive clinical details beyond hospitalization diagnoses, posing challenges in exploring syndrome associations. No data are available for the dispensation of over-the-counter medications, which largely underestimates the consumption of symptomatic pain-relieving drugs.

## 5. Conclusions

Using the National French Claim Database, we were able to demonstrate significant shifts in HRU associated with chronic pain management 6 months post discharge following COVD-19 hospitalization during the first pandemic wave. No distinct post-COVID-19 HRU pain patterns were identified. The observed changes are likely due to the pandemic character of exposure and aggressive management of disease symptoms during hospital stay. These variations in HRU are not significant enough to validate the concept of long COVID-19, at least for HRU in relation to patients’ pain, anxiety, and depression.

## Figures and Tables

**Table 1:** Studied endpoints of Health Resource Use.

Domain	Items
Physician consultations and rehabilitation	General Practice Neurology Rheumatology Physical Medicine Psychiatry Pain Medicine Non-coordinated visits Outpatient Physical Therapy Occupational therapy
Specific instrumental investigations	ENMG Nerve Sonography
Dispensed medications	Painkillers - Acetaminophen - NSAIDs - Weak opioids - Strong Opioids - Other analgesics Antidepressants with CPMA Antiepileptics with CPMA Antidepressants without CPMA Anxiolytics Hypnotics Vitamin D Midodrine
Hospitalizations	All-cause hospitalization Hospitalization for chronic pain

ENMG—Electroneuromyogram; CPMA—chronic pain modulation activity; NSAIDs—non-steroidal anti-inflammatory drugs.

**Table 2:** Chronic pain management-related domains and HRU in the overall population and in ICU survivors.

	HRU for All Patients, *n* = 68,822				HRU for Patients Discharged from ICU, *n* = 13,939			
	Before	After	HRU Ratio	HRU Rate Ratio CI 95%	Before	After	HRU Ratio	HRU Rate Ratio CI 95%
Medical visits								
General Practice	49,148 (71.4%)	51,014 (74.1%)	1.04	1.14 [1.13 to 1.16]	9900 (71.0%)	11,108 (79.7%)	1.12	1.13 [1.11 to 1.14]
Neurology	482 (0.7%)	487 (0.7%)	1.01	1 [0.94 to 1.07]	76 (0.6%)	107 (0.8%)	1.41	1 [0.98 to 1.02]
Rheumatology	1187 (1.7%)	959 (1.4%)	0.81	1 [0.94 to 1.06]	271 (1.9%)	212 (1.5%)	0.78	1 [0.98 to 1.02]
Physical Medicine	146 (0.2%)	109 (0.2%)	0.75	1.33 [1.08 to 1.65]	32 (0.2%)	30 (0.2%)	0.94	1 [0.9 to 1.11]
Psychiatry	1471 (2.1%)	1507 (2.2%)	1.02	1 [0.92 to 1.09]	286 (2.1%)	325 (2.3%)	1.14	0.95 [0.84 to 1.07]
Pain Medicine	35 (<0.1%)	30 (<0.1%)	0.86	1 [0.72 to 1.38]	11 (<0.1%)	11 (<0.1%)	1.00	1.35 [1.2 to 1.51]
Non-coordinated visits	472 (0.7%)	703 (1%)	1.49	1.33 [1.2 to 1.48]	85 (0.6%)	143 (1.0%)	1.68	1.13 [1.08 to 1.18]
Rehabilitation								
Outpatient Physical Therapy	34 (<0.1%)	2841 (4.1%)	83.56	0.95 [0.69 to 1.29]	11 (<0.1%)	1867 (13.4%)	169.73	0.99 [0.01 to 101.81]
Occupational therapy	822 (1.2%)	1517 (2.2%)	1.85	0.92 [0.81 to 1.05]	95 (0.7%)	601 (4.3%)	6.33	0.9 [0.17 to 4.73]
Technical acts								
ENMG	676 (1.0%)	1178 (1.7%)	1.74	1 [0.92 to 1.08]	11 (<0.1%)	1867 (13.4%)	169.73	1 [0.99 to 1.01]
Nerve Sonography	756 (1.1%)	880 (1.3%)	1.16	1 [0.92 to 1.08]	95 (0.7%)	601 (4.3%)	6.33	0.84 [0.81 to 0.86]
Medication Dispensing								
Acetaminophen	31,813 (46.2%)	33,537 (48.7%)	1.05	1.1 [1.08 to 1.12]	6228 (44.7%)	7158 (51.4%)	1.15	1.06 [0.83 to 1.36]
NSAIDs	12,511 (18.2%)	7412 (10.8%)	0.59	0.8 [0.78 to 0.82]	2697 (19.4%)	1365 (9.8%)	0.51	0.9 [0.89 to 0.92]
Weak opioids	9710 (14.1%)	9164 (13.3%)	0.94	0.88 [0.84 to 0.93]	2028 (14.6%)	2023 (14.5%)	1.00	0.97 [0.84 to 1.12]
Strong Opioids	1302 (1.9%)	2262 (3.3%)	1.74	0.88 [0.78 to 0.98]	214 (1.5%)	350 (2.5%)	1.64	0.91 [0.11 to 7.67]
Other analgesics	3981 (5.8%)	4430 (6.4%)	1.11	1.17 [1.06 to 1.28]	727 (5.2%)	961 (6.9%)	1.32	1.03 [0.87 to 1.21]
Antiepileptics with CPMA	4707 (6.8%)	5792 (8.4%)	1.23	0.9 [0.85 to 0.95]	842 (6.0%)	1356 (9.7%)	1.61	0.95 [0.88 to 1.03]
Antidepressants with CPMA	1924 (2.8%)	2117 (3.1%)	1.1	0.91 [0.88 to 0.94]	352 (2.5%)	445 (3.2%)	1.26	0.9 [0.8 to 1.02]
Antidepressants without CMPA	9266 (13.5%)	10,828 (15.7%)	1.17	0.93 [0.91 to 0.95]	1434 (10.3%)	1951 (14.0%)	1.36	0.97 [0.87 to 1.09]
Anxiolytics	11,952 (17.4%)	14,916 (21.7%)	1.25	0.92 [0.9 to 0.95]	2061 (14.8%)	2815 (20.2%)	1.37	0.92 [0.86 to 0.99]
Hypnotics	5436 (7.9%)	6759 (9.8%)	1.24	0.93 [0.89 to 0.97]	935 (6.7%)	1350 (9.7%)	1.44	0.93 [0.83 to 1.05]
Midodrine	163 (0.2%)	209 (0.3%)	1.28	1 [0.82 to 1.22]	12 (<0.1%)	34 (0.2%)	2.83	0.91 [0.45 to 1.85]
Hospitalizations								
All-reason hospitalizations	13,014 (18.9%)	19,809 (28.8%)	1.52	1.14 [1.08 to 1.21]	2445 (17.5%)	5045 (36.2%)	2.06	1 [0.97 to 1.03]
for Chronic pain	1437 (2.1%)	2154 (3.1%)	1.5	1 [0.94 to 1.07]	248 (1.8%)	442 (3.2%)	1.78	1.2 [1.18 to 1.21]

HRU—healthcare resource utilization; ICU—intensive care unit; CI—confidence interval; ENMG—Electroneuromyogram; CPMA—chronic pain modulation activity; NSAIDs—non-steroidal anti-inflammatory drugs.

## Data Availability

Data are not available due to legal restrictions.
